# Implications of Heterogeneity in Multiple Myeloma

**DOI:** 10.1155/2014/232546

**Published:** 2014-07-02

**Authors:** Sanjay de Mel, Su Hong Lim, Moon Ley Tung, Wee-Joo Chng

**Affiliations:** ^1^Department of Haematology-Oncology, National University Cancer Institute of Singapore, Singapore; ^2^Cancer Science Institute of Singapore, National University of Singapore, Singapore; ^3^National University Health System, NUHS Tower Block, Level 7, 1E Lower Kent Ridge Road, Singapore 119228

## Abstract

Multiple myeloma is the second most common hematologic malignancy in the world. Despite improvement in outcome, the disease is still incurable for most patients. However, not all myeloma are the same. With the same treatment, some patients can have very long survival whereas others can have very short survival. This suggests that there is underlying heterogeneity in myeloma. Studies over the years have revealed multiple layers of heterogeneity. First, clinical parameters such as age and tumor burden could significantly affect outcome. At the genetic level, there are also significant heterogeneity ranging for chromosome numbers, genetic translocations, and genetic mutations. At the clonal level, there appears to be significant clonal heterogeneity with multiple clones coexisting in the same patient. At the cell differentiation level, there appears to be a hierarchy of clonally related cells that have different clonogenic potential and sensitivity to therapies. These levels of complexities present challenges in terms of treatment and prognostication as well as monitoring of treatment. However, if we can clearly delineate and dissect this heterogeneity, we may also be presented with unique opportunities for precision and personalized treatment of myeloma. Some proof of concepts of such approaches has been demonstrated.

## 1. Introduction

Multiple myeloma (MM) is the second most common haematologic malignancies in the world. It arises from clonal plasma cells that secrete monoclonal proteins that can be measured in the serum and urine for diagnosis and disease monitoring. The disease manifests through anemia, hypercalcaemia, renal impairment, and lytic bone lesions. Patients may present with bone fractures, renal failure, and hence significant morbidity [1]. All myelomas are probably preceded by a precursor asymptomatic state called monoclonal gammopathy of undetermined significance (MGUS) or smoldering myeloma (SMM) [2, 3]. The progression to symptomatic disease is most likely through clonal evolution and acquisition of additional genetic events [4]. In recent years, a number of new treatments have been approved for myeloma, including thalidomide, bortezomib, lenalidomide, liposomal doxorubicin, carfilzomib, and pomalidomide. The increase in therapeutic options and the potency of these drugs have greatly improved the survival of patient who now survives for a median of 8 years from diagnosis [5]. Despite this progress, MM is still generally an incurable disease. Drug resistance and disease refractoriness are the common terminal pathways leading to death. A key factor underlying the clinical and therapeutic challenge is multiple layer of heterogeneity that exists in myeloma.

## 2. Molecular Heterogeneity

Studies over the years have shown that the MM genome is complex. However some of the genetic abnormalities cluster together which may suggest cooperating events. In addition, many abnormalities may affect similar pathways suggesting that there are key pathways affected in MM that may be important for disease pathogenesis and may represent good therapeutic targets.

### 2.1. Ploidy

At the chromosome level, myeloma can be broadly classified into hyperdiploid (48–74 chromosomes) and nonhyperdiploid myeloma. The hyperdiploid myeloma is characterized by a unique pattern of trisomies affecting many of the odd-numbered chromosomes such as chromosomes 3, 5, 7, 9, 11, 15, 19, and 21 [6]. The hyperdiploid and nonhyperdiploid dichotomy is an early event in myeloma pathogenesis as these patterns can be detected at the MGUS stage [7]. The reasons that this unique pattern of trisomies is seen and what triggers the acquisition of these trisomies are currently unknown.

### 2.2. Chromosome Gains and Losses

A number of chromosomal gains and deletions are common in multiple myeloma. These include deletion of chromosome 13, deletion of chromosome 17p13, deletion of chromosome 1p, and gain of chromosome 1q21. Chromosome 13 deletion is an early event that is also present in a substantial proportion of MGUS. Chromosome 17p13 deletion, chromosome 1p deletion, and gain of chromosome 1q21 on the other hand are most likely secondary events associated with disease progression, as they are rarely detected in MGUS [8]. Importantly, the critical gene(s) located on these chromosomes that may be of functional importance is not yet known. For chromosome 13, we previously showed that minimal deleted regions contain RB1 and NBEA and hence may be the implicated genes in this region [9]. In addition, miRNAs that are located within this region may also be relevant [10]. However, there is as yet no functional study that confirms their relevance to myeloma biology.

For chromosome 1q, a number of genes, such as CKS1B [11], BCL9 [12], MCL1 [13], PDZK1 [14], and MUC1 [15], have been implicated. However, it is still unclear whether one or more of these genes are relevant to myeloma biology. For chromosome 1p, we previously identified a region around 1p31-32, which is relevant for prognosis. Amongst the genes located within this locus, several have correlated expression with DNA copy number and may be functionally relevant, although there is no conclusive evidence so far [16]. For chromosome 17p13, the most likely candidate is* TP53*.* TP53* is within the minimally deleted region on chromosome 17p13 [17]. Furthermore, using sequencing in isolated plasma cells, p53 was mutated in 37% of patients with 17p13 deletion and none in those without [18]. However, 17p loss is almost always monoallelic and p53 mutation does not occur in most patients with 17p13 deletion, while methylation of the* TP53* promoter is relatively rare. This suggests that if p53 is the important gene, then it has to be acting in a haploinsufficient manner. We showed, using a panel of cell lines with different p53 abnormalities and using knockdown and overexpression studies, that the level of p53 affects its function and response to both genotoxic and nongenotoxic stress providing clear evidence that* TP53* is a haploinsufficieent tumor suppressor in MM (Teoh PJ et al. leukemia in press).

### 2.3. Translocations

These rearrangements juxtapose the strong promoters within the immunoglobulin heavy chain (IgH) gene locus to an oncogene and lead to very high expression of these oncogenes. A number of recurrent translocations have been identified in myeloma dysregulating a few classes of proteins, namely, FGFR3/MMSET, Cyclin D (Cyclin D1 and Cyclin D3), and MAFs [8] (Table 1). These are thought to be primary translocations as they already exist at the MGUS stage and are probably initiating events. In gene expression studies, these translocations drive cognate expression signatures that are dominant and are easily identified in gene expression studies. Besides these recurrent translocations, the IgH locus is also involved in nonrecurrent or what is sometimes called secondary translocations involving unknown partners. These are so called because they are thought to represent secondary events that may be important in disease progression.* MYC* is another gene that is recurrently rearranged. It was initially described as the archetypical genes involved in complex rearrangement in myeloma as a secondary event in myeloma progression. It was subsequently found that translocation of MYC can be detected in about 15% of newly diagnosed patient [19]. More recently, it was shown that the MYC pathway is activated in majority of myeloma patients and may be an important transforming event from MGUS to MM [20]. Using high resolution tiling arrays around the MYC locus, it was found that rearrangements of MYC are also common in newly diagnose myeloma and most of these rearrangements affect enhancers and superenhancers of MYC causing an increase in MYC expression [21].

### 2.4. Mutations

*RAS* was one of the commonly mutated genes in MM, detected in 20–45% of newly diagnosed myeloma patients depending on study cohort and methods used for mutation detection [6]. Both N-RAS and K-RAS can be mutated, with different studies showing different frequency of mutations in these genes. In a large ECOG study, mutations in* N-RAS* (about 70% of all* RAS* mutations) are more common than in* K-RAS* (about 30% of all RAS mutations) [22]. On the other hand, a study from the University of Arkansas found that the frequencies of* N-* and* K-RAS* mutations are quite similar [23]. Recently, sequencing studies in 203 MM patients found that* K-RAS* and* N-RAS* mutations are found in 23% and 20%, respectively [24]. RAS mutation is rare in MGUS [25]. This suggests that it is a potential transforming genetic factor and may also be involved in disease progression.* TP53*, a well-known tumor suppressor gene, is also mutated in myeloma although it is not very common. A large ECOG study using conformation sensitive gel electrophoresis found* TP53* mutated in 3% of cases [26]. More recently, mutation in* TP53* was found in 9% of cases using whole exome or whole genome sequencing. The use of deep sequencing has resulted in the identification of previously unknown genes that are recurrently mutated at a significant frequency in myeloma. These include* DIS3*,* FAM46C*,* BRAF*,* TRAF3*,* PRDM1*, and* RB1* (Table 2) [24].

### 2.5. Clustering

Amongst all these genetic chaos, some patterns and clustering around certain pathways exist. The recurrent primary translocations are predominantly seen in nonhyperdiploid myeloma. The primary events of translocations and hyperdiploidy seem to converge on the activation of one or more of the Cyclin D proteins [27].

Deletion 13 is also more common in nonhyperdiploid myeloma [28]. However, MYC rearrangements and activation occur more commonly in hyperdiploid myeloma. As MYC activation and chromosome 13 deletion are considered early events likely to play a role in disease transformation from MGUS to MM, it suggests that different genetic subtypes of myeloma may have different requirements for transformation. RAS mutations are not enriched according to ploidy but are significantly more common in tumors overexpressing Cyclin D1. More recent studies suggest that DIS3 mutations are significantly associated with nonhyperdiploid myeloma [24]. This suggests that there are specific combinations of genetic abnormalities that may collaborate during disease progression. On the other hand, secondary events such as IgH translocations involving unknown partners as well as chromosome 1 abnormalities and chromosome 17p13 deletions are distributed similarly across the main genetic subtypes.

Besides disease developmental pathways where there appears to be some preferential combination of abnormalities, overall, there are some pathways that are recurrently deregulated by different mechanisms in myeloma. The NFKB is affected through mutations, deletions, and amplifications of different genes including CYLD, TRAF3, TRAF2, and NFKB1A [24, 29, 30]. Cell cycle related genes are also commonly affected including CCND1, CCND2, CCND3, RB1, p16, and p18 [4, 23, 24]. Another commonly affected signaling pathway is the RAS-MAPK pathway with N-RAS, K-RAS, and BRAF recurrently mutated [24, 31]. Histone modifying enzymes, coagulation cascades, and the telomerase related pathways are also commonly affected (Table 3) [24, 31].

### 2.6. Clinical Implication

The clinical impact of these different genetic and molecular abnormalities is 3-fold. One, the different abnormalities has different prognostic relevance. Studies have consistently shown that deletion of 17p13 and the t(4;14) translocation whether detected by FISH or cytogenetics is an independent bad prognostic factor, although the adverse effect of t(4; 14) can be partially abrogated by velcade-based treatment [8, 32, 33]. Other abnormalities such as 1p21 amplification [34–36] and t(14; 16) [37–39] have also been commonly associated with poor outcome although this is not observed in every study. More recently a number of studies has shown that 1p deletion is independently and significantly associated with shorter survival, confirming earlier data from cytogenetic analysis [16, 40, 41]. Two, an understanding of the potential driving mutations in myeloma could lead to opportunities for precision medicine and targeted therapy. The recent description of a relapse patient with BRAF mutation responding spectacularly to BRAF inhibitor is one such proof of concept example [42]. It is therefore critical to identify driver mutations and pathways in myeloma so that patients can be matched to the most appropriate treatment. In this regard, treatment targeting the recurrently affect and functionally important pathway would be an important way forward. The cell cycle pathway, NFKB and RAS-MAPK pathways, would be good starting point. Third, some of these genetic mutations are predictors of response to drugs. In particular NFKB mutations, especially TRAF3 mutation, are associated with better response and progression free survival with bortezomib treatment [29]. More recently, mutation of NRAS but not KRAS has been shown to reduce myeloma sensitivity to bortezomib therapy [43].

## 3. Clinical Heterogeneity

### 3.1. Important Determinant of Outcomes

The variable outcome of patients with the same therapy suggests that there is underlying clinical heterogeneity. While some of this can be explained by the underlying biology of the tumor as described in the sections above, other factors such as host factors and extent of disease involvement are also important.

Age is an important prognostic factor. It impacts on the ability of patient to tolerate treatment and cope with disease complications. A large global study showed that there is progressive shortening of survival with increasing age even in patients treated with novel agents [44]. Conversely, a young age is an important independent factor associated with very long outcome [45].

A number of factors that may reflect underlying disease burden have been developed. The Durie-Salmon staging system combines a number of factors including renal function and number of bone lesions [46]. However, the system is cumbersome and lacks sensitivity. It has since been superceded by the International Staging System which is made up of 2 factors, beta-2 microblobulin, which reflects disease activity, and albumin, which reflects host fitness. The ISS is robustly derived and validated in large global cohorts and is easy to apply. These 2 factors emerged as the most significant and independent prognostic factors, trumping more traditional prognostic factors such as blood counts, m-protein levels, bone marrow plasma cell involvement, renal function, calcium level, and the types of immunoglobulin [47]. The incorporation of ISS with high-risk genetics provides further refinement to the prognostic system [48].

Can we further refine this dissection of clinical heterogeneity to more accurately segregate patients with different response to treatment and outcome? Gene expression profiles are very strong predictors of outcome. There are a number of signatures that are independently associated with poor outcome (Table 4). However, the confusion surrounds which one is best, how best to use them, and why they hardly share any individual genes. We undertook a meta-analysis to look at these signatures individually and in combination to set up a framework for applying gene expression signature for the prognosis of patients in the clinical setting. We found that combinations of signatures are more powerful predictors of outcome than any individual signature and we develop a package, which will be available to all investigators to apply to their own data for real-time prospective validation (Chung TH et al. in press).

The next challenge will be to assess this GEP signature in combination with ISS and high-risk genetics in an international exercise along the lines of ISS to hopefully arrive at a unified prognostic system.

### 3.2. Therapeutic Implications

The ability to carefully dissect the risk of patients in relation to the different treatment regimen and strategy is important. While the path towards precision medicine in myeloma will be a long process as we have to develop the treatment to match to patients' different mutations and also importantly identify the critical mutations to target, what we already have now are very effective treatments that have benefitted a large number of patients. How best to use the current treatment in terms of optimizing benefit while minimizing unnecessary treatment and toxicity is therefore a highly relevant clinical questions. Based on currently available prognostic factors, patients can be stratified into 3 risk groups. While at present, there is no evidence that different treatment should be recommended for the different risk groups, it will be important for risk stratification to be incorporated into future trial design to ensure that we can optimize treatment according to risk groups [57].

## 4. Clonal Heterogeneity

Data from next generation sequencing studies has challenged the traditional dogma that clonal evolution in cancer occurs in a linear fashion through stepwise accumulation of mutations. Numerous studies now propose a branching pattern of clonal evolution in keeping with Darwinian principles [58]. In myeloma it is no different. Furthermore, these studies suggest related clones with different composition of genetic abnormalities coexist.

### 4.1. Evidence for Clonal Heterogeneity and Evolution in MM

Recent studies using single nucleotide polymorphism (SNP) arrays and whole exome sequencing [59], array comparative genomic hybridization (aCGH) [60], and whole genome sequencing (WGS) [61] have clearly demonstrated the presence of multiple clonally related tumor cell populations within the same patient that has different composite of genetic aberrations.

In a clinical study using SNP arrays and whole exome sequencing on 67 MM patients, the percentage of subclonal mutations in each patient varied from 10% to 80% [59]. In a study using WGS on bone marrow samples obtained from a patient with MM at serial time points from diagnosis to demise, 15 mutations were found to be present at all time points, representing the mutational profile of the common ancestor clone. The samples taken at each relapse had unique mutations, some of which disappeared at the first relapse and returned at subsequent relapse [61]. These findings strongly support the hypothesis that clonal evolution occurred in this patient and contributed to her disease progression; it also raises the possibility of alternating clonal dominance that may be selected under treatment pressure.

Using aCGH in 28 patients with serial samples, Keats et al. identified three patterns of clonal evolution among the patients: loss of copy number abnormalities (CNA), gain of CNA, and both losses and gains of CAN, with increasing number of CNAs as the disease progress. There is also an association between increase CAN and high-risk genetic abnormalities. In a patient whom they have material to study in depth, they were able to identify four clones that showed alternating dominance at different time points. In addition, specific clones were selected for by proteasome inhibitor treatment and melphalan. Clonal dominance and selection by treatment is further demonstrated using a mouse model of myeloma [60].

### 4.2. Mechanisms of Clonal Evolution

Clonal evolution occurs due to driver mutations or larger scale genomic crises, which can be stimulated by tumour, host, environmental, and treatment-related factors. A number of driver mutations have been implicated in the clonal evolution of MM (Table 5). Chromothripsis is an example of a genomic crisis, which is characterized by spontaneous catastrophic chromosome breakage followed by reassembly resulting in significant loss of chromosomal material [62]. Features of chromothripsis were detected in 1.3% of patients in a study of 764 patients with newly diagnosed MM. Of the 10 patients with chromothripsis, five relapsed within one year and three died during that period [63]. This study suggests that chromothripsis is associated with rapid clinical progression and a poor prognosis that needs to be validated in further studies.

It has recently been shown by whole exome sequencing that clonal heterogeneity is also present at the asymptomatic MGUS and SMM stages. The investigators went on to demonstrate that the majority of subclones present at the MM stage are already present at the SMM stage. Genetic lesions that may trigger the transformation from SMM to MM were identified [64] (Table 5).

### 4.3. Clinical Implications of Clonal Heterogeneity

The emerging concept of clonal heterogeneity and alternating clonal dominance in myeloma has clear therapeutic implications. In the patient studied by Keats et al., the dominant clone detected during her first relapse had mutations leading to NFkB activation, this clone was effectively treated with carfilzomib. At her second relapse however the dominant clone was associated with a different mutational profile and she had an inferior response to bortezomib [60]. These findings suggest that we should have an understanding of the clonal composition of the tumor at each treatment phase and use this information to guide treatment. The challenge is to identify treatment modalities and combinations effective against the main functionally relevant genetic abnormalities in MM. While the current treatment armamentarium is expanding, it is still limited to a few classes of drugs. We need to expand this portfolio in a systematic manner to match treatment with mutational profiles. The use of patient-derived xenograft models and faithful myeloma mouse models such as the V∗K-MYC model [65] and the availability of an increasingly broad range of novel therapeutics should facilitate the generation of this knowledge.

## 5. Cellular Differentiation Heterogeneity

Evidence is emerging that the clonal cells that constitute myeloma are not homogenously of mature plasma cell phenotype. In fact, a hierarchy of precursor cells with different clonogenic potential, different gene expression, phenotype, and sensitivity to therapy may exist. Sitting at the apex of this hierarchy of clonally related cellular population is the putative clonogenic myeloma progenitor cells (MPC).

### 5.1. Evidence for the Existence of MPC

MM plasma cells are quiescent and have a low proliferative index. As a result, its tumorigenic potential and ability has been questioned [66]. It is therefore postulated that a precursor population might be responsible for disease initiation.

Early work by Hamburger and colleagues showed that bone marrow cells from MM patients were capable of in vitro colony formation [67]. Bakkus and coworkers later identified a population of mature B-cells in the peripheral blood and marrow of MM patients that were clonally related to MM plasma cells and hence postulated to be MPCs [68]. In a separate report, a patient with MM, who went on to developed B lymphoblastic leukaemia, has B lymphoblasts that share similar clonal immunoglobulin gene rearrangement as the malignant PCs detected at diagnosis. The leukaemic B-cells when grafted into NOD/SCID mice led to the development of lytic bone lesions. The B-cells identified in the bone marrow of these mice were CD45+, CD19+, CD20+, and CD138−. This study suggested that some of the circulating B-cells in the patient were MPCs capable of initiating MM in vivo [69].

More recently, Matsui et al. isolated CD138− mature B-cells from MM cell lines as well as clinical samples. This population had a greater capacity for colony formation in vitro and in vivo than CD138+ MM plasma cells and their expression of Ki 67 was greater [70]. They also showed that these MM forming mature B-cells had a CD19+, CD20+, and CD138− phenotype with light chain restriction, consistent with the phenotype of the putative MM clonogenic cells identified in earlier studies [71].

The expression of aldehyde dehydrogenase (ALDH) is characteristic of cancer stem cells [72]. Using ALDH as a marker, Reghunathan and coworkers identified a CD138− population constituting 2.5% of cells in a human MM cell line. Indeed, 45% of the CD138− population expresses ALDH compared to less than 1% of the CD138+ cells. These CD138−ALDH+ cells had superior colony forming capacity both in vitro and in vivo. They also have similar gene expression profiles compared to hematopoietic stem cells (HSC) and leukaemic stem cells (LSC). These data further support the existence of a clonogenic population in the CD138− fraction of MM [73].

### 5.2. Hierarchical Organization of MPC

Clonally related subpopulations of MPCs, sharing common IgH gene rearrangement, have been detected in bone marrow of MM patients. Amongst these immunophenotypically distinct subpopulations, the CD19+CD138− cells had a superior colony forming capacity compared to the CD138+ population [75]. Chaidos and colleagues analyzed bone marrow samples from 10 MM patients and identified four clonally related subpopulations of MPC. Using a mathematical growth model they showed that differentiation occurred in the following sequence: memory B-cell followed by plasmablast followed by preplasma cell and finally plasma cell. Importantly, they demonstrated that the CD138− dim plasma cells were also capable of reverting back to a preplasma cell phenotype [76]. A hierarchical organization of clonally related MPCs was furtherly demonstrated by studying the expression of XBP-1, a transcription factor important for the differentiation of plasma blasts into plasma cells. In this study, five clonally related subpopulations of MPCs in bone marrow samples of MM patients were identified with XBP-1 expression universally negative in the CD38−CD138− populations [77].

These studies strongly suggest the presence of a clonal hierarchy of MPCs consisting of phenotypically distinct subsets with a defined maturation sequence (see Table 6).

### 5.3. Gene Expression Differs between MPC and Mature Plasma Cells

Several studies have compared the gene expression profile of CD138− to CD138+ clonal myeloma cells. In 2 of these studies [73, 76], the polycomb repressor complex 2 (PRC2) related genes, such as EZH2, EED, and SUZ12, were upregulated in the CD138− subsets. The PRC genes promote histone methylation and reduced expression of their targets. The overexpression of PRC2 genes and subsequent reduced expression of target genes such as the cyclin dependent kinase inhibitors (such as p21) would increase the proliferative capacity of the CD138− MPCs. In one of these studies, other genes involved in epigenetic regulation of gene expression such as histone demethylases, histone acetyltransferases, and deacetylases also have altered expression. The resultant “epigenetic plasticity” may explain the bidirectional transition of MPCs observed in the study [76]. In another study, the RAR*α*2 gene was found to be overexpressed in the CD138− subset. Overexpression of RAR*α*2 resulted in the activation of the Wnt and Hedgehog pathways, increased expression of ALDH, expression of embryonic stem cell genes, and greater clonogenic potential in the MM cells. These effects were reversed upon silencing of RAR*α*2 [78].

Two groups have also looked at the clinical relevance of gene expression signature derived from these clonogenic myeloma cells. Kassambara and colleagues focused on genes differentially expressed in clonogenic myeloma cells that are not related to proliferation or previous prognostic gene expression signatures. They identified 50 genes which were of prognostic significance in MM. Thirty-seven of these 50 genes were also found to be overexpressed in three human stem cell populations, pluripotent stem cells, hematopoietic stem cells, and mesenchymal stem cells. They went on to build a “stem cell score” based on the expression of these genes, which proved strongly prognostic in two independent patient cohorts [79]. In the study of Reghunathan and colleagues, the gene signature comprising genes differentially expressed in CD138− clonogenic population compared to the CD138+ population was associated with poorer outcome in stem cell transplant as well as velcade-treated MM patient cohorts.  Table 7 summarizes the important genes differentially expressed between MPC and mature PC.

### 5.4. Clinical Significance of MPC

#### 5.4.1. Implications for Minimal Residual Disease (MRD) Assessment

**Figure 1:**
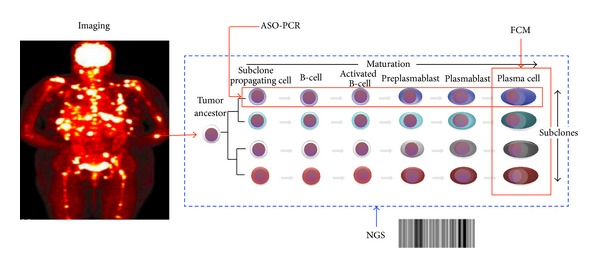
Implications of heterogeneity on MRD detection. MM involvement may be patchy and involve extramedullary sites. All these lesions may be detected by whole body imaging modality such as PET-CT scan. Within the individual lesions, 2 dimensions of heterogeneity may exist in the population of tumor cells. On one hand, there may be clonal heterogeneity where related clones with different genetic composition may coexist. On the other hand, clonally related progenitor populations at earlier stage of differentiation may exist. Flow cytometry can detect the plasma cell component but not the precursor population while ASO-PCR can detect all the clonal cells including the precursor population but its applicability is limited. The development of NGS methods may allow utility in larger population of patients.

Recent studies have shown the importance of MRD, as assessed by flow cytometry (FC) [80, 81], allele specific oligonucleotide (ASO) PCR [82], or PET imaging [83], in predicting early relapse and poorer outcome in patients who have achieve conventional complete remission as defined by the International Myeloma Working Group. While potentially clinically useful, each technique has its technical advantages and limitations but importantly they also assess different tumor cell populations of the disease. As a result, the recent insights into the hierarchical organization of clonally related myeloma precursors need to be taken into consideration when we choose the techniques to utilize. Both the FC and PCR methods only assess the bone marrow tumor cells as these tests are performed on bone marrow samples. Therefore, disease outside the bone marrow or even disease within the bone marrow, that is, not in the area sampled, may be missed for MRD assessment. This limitation is relevant as studies have shown that the CD138− clonogenic MPCs have propensity for extramedullary sites [76]. This is where whole body imaging such as PET-CT may be useful in identifying extramedullary disease. However, the sensitivity of PET-CT in terms of MRD assessment is still unclear. Comparing FC and PCR, there is also subtle yet important difference in the population they may detect. FC-based MRD assessment is based on the detection of the aberrant phenotype of myeloma plasma cells and is therefore predominantly detecting the plasma cell component and hence will miss the clonogenic MPCs. PCR on the other hand detects the clonal rearrangements of IgH gene and therefore will identify any clonally related cells, including the MPCs (Figure 1). The predominant limitation of the PCR method is that clone specific rearrangements can only be identified in only less than 50% of cases in MM. This limitation may be overcome by using sequencing-based method to detect IgH gene rearrangement, which can be applied to more than 90% of cases (Martinez-Lopez et al. blood* in press*). Due to the challenge of cellular heterogeneity in MM, it is likely that more than 1 technique will need to be used for comprehensive MRD assessment in the future. Prospective study should be conducted to correlate PET-CT with FC and PCR/sequencing-based methods and to develop clinically useful algorithm for the application of these techniques possibly in a step-wise manner.

### 5.5. The Role of MPC in Drug Resistance and Disease Relapse

CD138− clonogenic MPCs have been shown by a number of groups to be more resistant to drugs used for myeloma treatment such as lenalidomide, dexamethasone, and bortezomib. This is in part due to the increased expression of ABCG2/BCRP drug transporter as well as higher levels of ALDH [84]. The expression of these drug transporters may be induced by an increased expression of RAR*α*2 [78]. More recently, through a series of elegant experiments, it was shown that in Velcade resistant patients, there is an increase in XBP1 low expressing MPCs which share common genetic changes as the myeloma plasma cells. These cells produce less immunoglobulin and have less endoplasmic reticulum (ER) stress and express less unfolded protein response (UPR) genes and are therefore more resistant to velcade, which induce cell death in plasma cells with high immunoglobulin production and ER stress by inhibiting the UPR, which requires an active proteasome [77].

MPCs have also been shown to rely on survival and proliferation pathways used by stem cells. Peacock and colleagues demonstrated that CD138− CD19+ B-cells from MM cell lines and patient samples had constitutive activation of the Hedgehog (Hh) signaling pathway evident by the increased expression of the smoothened (SMO) protein [85]. It is possible that MPCs rely on these to avoid cell death induced by agents that are active against mature plasma cells.

### 5.6. Targeted Therapy against MPCs

Signaling pathways and other lesions unique to the clonogenic MPCs may form the basis for targeted therapy against MPCs. The histone methyltransferase inhibitor DZNep inhibits PRC2 and was shown to be more effective in killing CD138− compared to CD138+ MM cells [73]. Inhibition of Hh pathway signaling by cyclopamine was shown to reduce the clonogenic capacity of the CD138−CD19+ MPC fraction in two MM cell lines [85]. A phase I trial using another Hh pathway antagonist GDC-0449 (Vimodegib) in patients with high risk MM postautologous stem cell transplant has been completed and results are awaited. Blockade of the JAG-NOTCH interaction using NOTCH-Fc chimeric molecules resulted in impaired self-renewal capacity in MM cell lines [86]. Targeting the NOTCH pathway using the NOTCH inhibitor R0490927 in combination with melphalan has also been investigated in a phase II clinical trial for which the results are awaited. Based on the identification of high RAR*α*2 expression in the CD138− MPC, Yang and coworkers showed that al-trans retinoic acid (ATRA) preferentially induced apoptosis in the CD138− fraction [78]. Telomerase activity is required for the survival of normal stem cells. Brennan and colleagues treated MM stem cells from cell lines and clinical samples with the telomerase inhibitor imetelstat. They found that telomerase inhibition resulted in inhibition of clonogenic growth as well as reduced expression of genes expressed by stem cells [87]. Clinical trials using telomerase inhibitors in MM are awaited. The long-term remissions achieved by selected MM patients who survive allogeneic stem cell transplant suggest the presence of a graft versus myeloma stem cell effect [88]. This has led to investigation of cellular therapy modalities targeting MPCs. Swift and colleagues demonstrated that the natural killer (NK) cell lines KHYG2 and NK-92 were selectively toxic to the MPC fraction in vitro [89]. Clinical trials using NK cells in MM are in progress. The concepts are still in early phase of clinical development and results from early phase clinical trials are still pending (Table 8).

## 6. Conclusion

Multiple levels of heterogeneity exist in MM, providing tremendous clinical challenges in diagnosis, prognosis, treatment, and monitoring. The understanding of biological relevance of the heterogeneity at the molecular, clonal, and cellular level and how these relate to clinical heterogeneity will provide important mechanistic insights that will guide future development of diagnostic, prognostic, therapeutic, and monitoring modalities to further personalize treatment, improve treatment precision, and lengthen the survival of MM patients.

## Figures and Tables

**Table 1:** Recurrent translocations involved in multiple myeloma.

Translocations	Gene deregulated	Frequency
t(4; 14) (p16; q32)	MMSET FGFR3	15%
t(11; 14) (q13; q32)	CCND1	16%
t(6; 14) (p21; q32)	CCND3	2%
t(12; 14) (p13; q32)	CCND2	<1%
t(14; 16) (q32; q23)	MAF	5%
t(14; 20) (q32; q11)	MAFB	2%
t(8; 14) (q24; q32)	MAFA	1%

**Table 2:** Genes affected by recurrent mutations in multiple myeloma.

Gene	Frequency (%)
KRAS	23
NRAS	20
DIS3	11
FAM46C	11
TP53	8
BRAF	6
TRAF3	5
PRDM1	5
RB1	3
CYLD	2

**Table 3:** Pathways commonly affected by mutations.

Pathway	Mutated genes
Cell cycle pathway including G1-S phase transition and checkpoints	CCNA1, CCNB1, CCND1, CDK4, CDK6, CDK7, CDKN1B, CDKN2A, CDKN2C, RBL1, CDK4, PRB1, ABL1, ATM, ATR, CDK6, SKP2, TGFB1, TGFB2, TGFB3

TERT pathway	MAX, MYC, SP1, SP3, WT1

p38 MAPK pathway	ATF2, DAXX, GRB2, HMGN1, MAP2K6, MAP3K7, MAP3K9, MAPK14, MAX, MEF2A, MEF2D, MKNK1, MYC, PLA2G4A, RAC1, RIPK1, RPS6KA5, SHC1, TGFB1, TGFB2, TGFB3, TRAF2

Histone methyltransferase	KDM6A, MLL, MLL2, MLL3, NSD1, WHSC1, WHSC1L1

NFKB pathway	BIRC2, BIRC3, BTRC, CARD10, CARD11, CARD6, CARD8, CYLD, FBXW11, IKBIP, IKBKAP, IKBKB, IKBKE, IL1R1, IRAK1, MAP3K14, MAP3K7, MYD88, NFKB2, NFKBIB, NOD2, RELA, RIPK1, RIPK2, RIPK4, TLR4, TRAF2, TRAF3, TRAF3IP1

Clotting pathway	COL4A1, COL4A2, COL4A3, COL4A5, COL4A6, F11, F3, F5, F7, F8, FGA, FGG, TFPI

**Table 4:** GEP-based prognostic signatures.

GEP signature	Methods
UAMS 70-gene [49]	Derived by comparing expression of profiles of patients with top and bottom quartile of survival treated on total therapy II

IFM signature [50]	Derived by comparing expression of profiles of patients with good and poor outcome in IFM trials

Centrosome index [51]	Based on expression of constituents of the centrosome

HZD cell death signature [52]	Signature derived from genes homozygously deleted in myeloma as detected by array comparative genomic hybridization

IL6-HMCL signature [53]	13-gene signature from genes induced upon IL6 stimulation of human myeloma cells lines

Proliferation index [54]	Curated signature based on proliferation genes

EMC 92-gene signature [55]	92-gene signature based on differentially expressed genes between patient with good and poor outcome on HOVON trial

Chromosome instability genomic event count (CINGEC) signature [56]	Based on differentially expressed genes between patients with the top and bottom quartile of genomic instability score based on number of genetic abnormalities identified by array comparative genomic hybridization

**Table 5:** Driver mutations that may be responsible for clonal evolution in MM.

Affected gene	Type of mutation	Normal function	Postulated role in disease evolution	Study
AFF1	Damaging	Histone methylation	Driver of Myelomagenesis (found at all disease time points)	Egan et al. 2012 [61]
RUNX2	Inactivating	Regulates osteopontin a bone matrix glycoprotein involved in cell survival	HR SMM to MM	Walker et al. 2014 [64]
BRCA2	Disrupted due to t(13; 21)	DNA repair	HR SMM to MM	Walker et al. 2014 [64]
UNC5D	Inactivating	Induces apoptosis, regulated by p53	HR SMM to MM	Walker et al. 2014 [64]
ZKSCAN3	Truncating	Possible effects on VEGF	PCL transformation	Egan et al. 2012 [61]
Rb1	Truncating	Key tumour suppressor gene	PCL transformation	Egan et al. 2012 [61]

VEGF = Vascular endothelial growth factor, PCL = Plasma cell leukaemia, and HR SMM = High risk smouldering multiple myeloma.

**Table 6:** Subtypes of MPC and their phenotypes described in the key publications.

Study	MPC subpopulation and phenotype				
Rasmussen 2000 [74]	*Early B cell* CD38+, CD19+	*More differentiated B cell* CD38−, CD19+			
Boucher et al. 2012 [75]	*LCR B-cells* CD138−/CD34+/CD19+	*More Differentiated B cells* CD138−/CD34−/CD19+	*Plasma cells* CD138+/CD34−/CD19−		
Chaidos et al. 2013 [76]	*Memory B cells* CD 19+, CD 138−, CD 38−	*Plasmablasts* CD 19+, CD 138−, CD38+	*Preplasma cells * ** ** CD 19−, CD 138−, CD38+, CD56+	*Plasma cells* CD 19−, 138dim or +, CD 56+, CD38	
Leung-Hagesteijn et al. 2013 [77]	*B cell* CD20+, CD38−, CD138−	*Activated B-cell* CD20low, CD38−, CD138−	*Preplasmablast* CD20−, CD38−, CD138−	*Plasmablast* CD20−, CD38+, CD138+/−	*Plasma cell* CD20−, CD38+, CD138+

LCR = Light chain restricted.

**Table 7:** Summary of differential gene expression between CD 138− and CD 138+ subsets. The list of genes is not exhaustive but includes selected genes of importance described in the studies.

Study	Gene	Differential expression	Function
Reghunathan et al. 2013 [73]	PRC2 related (EZH2, EED, SUZ12) PRC1 related (BMI1)	Upregulated in CD 138− subset.	Via histone methylation, reduces the expression of p21 and other CDK inhibitors, driving proliferation.
	BMP 2, BMP3, BMP4	Upregulated in CD 138+ subset	Promote differentiation of plasmablasts to mature plasma cells.

Yang et al. 2013 [78]	RAR*α*2	Upregulated in CD 138− subset.	Increased ALDH expression, increased activity of WNT and Hedgehog pathway signaling as well as Cyclin D1.
	Oct 4, SOX 2, Nanog, Lin 28A	Upregulated in CD 138− subset	Genes expressed in pluripotent stem cells.

**Table 8:** Summary of agents in development which may be selectively toxic to MPC.

Drug/molecule	Mechanism of action	Phase/used in combination with other agents
DZNep inhibitor	Disruption of PRC2	Preclinical
Vimodegib (GDC-0449)	Hedgehog signaling inhibitor	Phase I/after auto SCT
R0490927	NOTCH signaling inhibitor	Phase II/melphalan
MK571	MRP3 inhibitor	Preclinical/bortezomib
ATRA	Induces degradation of RAR*α*2	Preclinical
Imetelstat	Telomerase Inhibitor	Preclinical
NK cell therapy	Cellular cytotoxicity	Preclinical

SCT = Stem cell transplant.

## References

[1] Kyle RA, Rajkumar SV (2008). Multiple myeloma. *Blood*.

[2] Weiss BM, Abadie J, Verma P, Howard RS, Kuehl WM (2009). A monoclonal gammopathy precedes multiple myeloma in most patients. *Blood*.

[3] Landgren O, Kyle RA, Pfeiffer RM (2009). Monoclonal gammopathy of undetermined significance (MGUS) consistently precedes multiple myeloma: a prospective study. *Blood*.

[4] Morgan GJ, Walker BA, Davies FE (2012). The genetic architecture of multiple myeloma. *Nature Reviews Cancer*.

[5] Ocio EM, Richardson PG, Rajkumar SV (2014). New drugs and novel mechanisms of action in multiple myeloma in 2013: a report from the International Myeloma Working Group (IMWG). *Leukemia*.

[6] Fonseca R, Barlogie B, Bataille R (2004). Genetics and cytogenetics of multiple myeloma: a workshop report. *Cancer Research*.

[7] Chng WJ, van Wier SA, Ahmann GJ (2005). A validated FISH trisomy index demonstrates the hyperdiploid and nonhyperdiploid dichotomy in MGUS. *Blood*.

[8] Fonseca R, Bergsagel PL, Drach J (2009). International Myeloma Working Group molecular classification of multiple myeloma: spotlight review. *Leukemia*.

[9] O’Neal J, Gao F, Hassan A (2009). Neurobeachin (NBEA) is a target of recurrent interstitial deletions at 13q13 in patients with MGUS and multiple myeloma. *Experimental Hematology*.

[10] Roccaro AM, Sacco A, Thompson B (2009). MicroRNAs 15a and 16 regulate tumor proliferation in multiple myeloma. *Blood*.

[11] Zhan F, Sawyer J, Gupta S (2004). Elevated expression of *CKS1B* at 1q21 is highly correlated with short survival in myeloma. *Blood*.

[12] Willis TG, Zalcberg IR, Coignet LJA (1998). Molecular cloning of translocation t(1;14)(q21;q32) defines a novel gene (BCL9) at chromosome 1q21. *Blood*.

[13] Craig RW, Jabs EW, Zhou P (1994). Human and mouse chromosomal mapping of the myeloid cell leukemia-1 gene: MCL1 maps to human chromosome 1q21, a region that is frequently altered in preneoplastic and neoplastic disease. *Genomics*.

[14] Inoue J, Otsuki T, Hirasawa A (2004). Overexpression of PDZK1 within the 1q12-q22 amplicon is likely to be associated with drug-resistance phenotype in multiple myeloma. *American Journal of Pathology*.

[15] Dyomin VG, Palanisamy N, Lloyd KO (2000). MUC1 is activated in a B-cell lymphoma by the t(1;14)(q21;q32) translocation and is rearranged and amplified in B-cell lymphoma subsets. *Blood*.

[16] Chng WJ, Gertz MA, Chung T-H (2010). Correlation between array-comparative genomic hybridization-defined genomic gains and losses and survival: identification of 1p31-32 deletion as a prognostic factor in myeloma. *Leukemia*.

[17] Boyd KD, Ross FM, Tapper WJ (2011). The clinical impact and molecular biology of del(17p) in multiple myeloma treated with conventional or thalidomide-based therapy. *Genes Chromosomes and Cancer*.

[18] Lodé L, Eveillard M, Trichet V (2010). Mutations in TP53 are exclusively associated with del(17p) in multiple myeloma. *Haematologica*.

[19] Avet-Loiseau H, Gerson F, Magrangeas F, Minvielle S, Harousseau J-L, Bataille R (2001). Rearrangements of the c-myc oncogene are present in 15% of primary human multiple myeloma tumors. *Blood*.

[20] Chng W-J, Huang GF, Chung TH (2011). Clinical and biological implications of MYC activation: a common difference between MGUS and newly diagnosed multiple myeloma. *Leukemia*.

[21] Affer M, Chesi M, Chen WD (2014). Promiscuous rearrangements of the MYC locus hijack enhancers and super-enhancers to dysregulate MYC expression in multiple myeloma. *Leukemia*.

[22] Chng WJ, Gonzalez-Paz N, Price-Troska T (2008). Clinical and biological significance of RAS mutations in multiple myeloma. *Leukemia*.

[23] Chng WJ, Glebov O, Bergsagel PL, Kuehl WM (2007). Genetic events in the pathogenesis of multiple myeloma. *Best Practice and Research: Clinical Haematology*.

[24] Lohr JG, Stojanov P, Carter SL (2014). Widespread genetic heterogeneity in multiple myeloma: implications for targeted therapy. *Cancer Cell*.

[25] Rasmussen T, Kuehl M, Lodahl M, Johnsen HE, Dahl IMS (2005). Possible roles for activating RAS mutations in the MGUS to MM transition and in the intramedullary to extramedullary transition in some plasma cell tumors. *Blood*.

[26] Chng WJ, Price-Troska T, Gonzalez-Paz N (2007). Clinical significance of TP53 mutation in myeloma [13]. *Leukemia*.

[27] Bergsagel PL, Kuehl WM, Zhan F, Sawyer J, Barlogie B, Shaughnessy J (2005). Cyclin D dysregulation: an early and unifying pathogenic event in multiple myeloma. *Blood*.

[28] Fonseca R, Debes-Marun CS, Picken EB (2003). The recurrent IgH translocations are highly associated with nonhyperdiploid variant multiple myeloma. *Blood*.

[29] Keats JJ, Fonseca R, Chesi M (2007). Promiscuous mutations activate the noncanonical NF-*κ*B pathway in multiple myeloma. *Cancer Cell*.

[30] Annunziata CM, Davis RE, Demchenko Y (2007). Frequent engagement of the classical and alternative NF-*κ*B pathways by diverse genetic abnormalities in multiple myeloma. *Cancer Cell*.

[31] Chapman MA, Lawrence MS, Keats JJ (2011). Initial genome sequencing and analysis of multiple myeloma. *Nature*.

[32] Munshi NC, Anderson KC, Bergsagel PL (2011). Consensus recommendations for risk stratification in multiple myeloma: report of the International Myeloma Workshop Consensus Panel 2. *Blood*.

[33] Avet-Loiseau H (2007). Role of genetics in prognostication in myeloma. *Best Practice and Research: Clinical Haematology*.

[34] Hanamura I, Stewart JP, Huang Y (2006). Frequent gain of chromosome band 1q21 in plasma-cell dyscrasias detected by fluorescence in situ hybridization: incidence increases from MGUS to relapsed myeloma and is related to prognosis and disease progression following tandem stem-cell transplantation. *Blood*.

[35] Fonseca R, van Wier SA, Chng WJ (2006). Prognostic value of chromosome 1q21 gain by fluorescent in situ hybridization and increase CKS1B expression in myeloma. *Leukemia*.

[36] Avet-Loiseau H, Attal M, Moreau P (2007). Genetic abnormalities and survival in multiple myeloma: the experience of the Intergroupe Francophone du Myélome. *Blood*.

[37] Avet-Loiseau H, Malard F, Campion L (2011). Translocation t(14;16) and multiple myeloma: is it really an independent prognostic factor?. *Blood*.

[38] Boyd KD, Ross FM, Chiecchio L (2012). A novel prognostic model in myeloma based on co-segregating adverse FISH lesions and the ISS: analysis of patients treated in the MRC Myeloma IX trial. *Leukemia*.

[39] Fonseca R, Blood E, Rue M (2003). Clinical and biologic implications of recurrent genomic aberrations in myeloma. *Blood*.

[40] Hebraud B, Leleu X, Lauwers-Cances V (2014). Deletion of the 1p32 region is a major independent prognostic factor in young patients with myeloma: the IFM experience on 1195 patients. *Leukemia*.

[41] Boyd KD, Ross FM, Walker BA (2011). Mapping of chromosome 1p deletions in myeloma identifies FAM46C at 1p12 and CDKN2C at 1p32.3 as being genes in regions associated with adverse survival. *Clinical Cancer Research*.

[42] Andrulis M, Lehners N, Capper D (2013). Targeting the BRAF V600E mutation in multiple myeloma. *Cancer Discovery*.

[43] Mulligan G, Lichter DI, di Bacco A (2014). Mutation of NRAS but not KRAS significantly reduces myeloma sensitivity to single-agent bortezomib therapy. *Blood*.

[44] Ludwig H, Bolejack V, Crowley J (2010). Survival and years of life lost in different age cohorts of patients with multiple myeloma. *Journal of Clinical Oncology*.

[45] Avet-Loiseau H, Attal M, Campion L (2012). Long-term analysis of the ifm 99 trials for myeloma: cytogenetic abnormalities [t(4;14), del(17p), 1q gains] play a major role in defining long-term survival. *Journal of Clinical Oncology*.

[46] Durie BGM, Salmon SE (1975). A clinical staging system for multiple myeloma. Correlation of measured myeloma cell mass with presenting clinical features, response to treatment, and survival. *Cancer*.

[47] Greipp PR, Miguel JS, Dune BGM (2005). International staging system for multiple myeloma. *Journal of Clinical Oncology*.

[48] Avet-Loiseau H, Durie BGM, Cavo M (2013). Combining fluorescent in situ hybridization data with ISS staging improves risk assessment in myeloma: an International Myeloma Working Group collaborative project. *Leukemia*.

[49] Shaughnessy JD, Barlogie B (2006). Using genomics to identify high-risk myeloma after autologous stem cell transplantation. *Biology of Blood and Marrow Transplantation*.

[50] Decaux O, Lodé L, Magrangeas F (2008). Prediction of survival in multiple myeloma based on gene expression profiles reveals cell cycle and chromosomal instability signatures in high-risk patients and hyperdiploid signatures in low-risk patients: a study of the Intergroupe Francophone du Myélome. *Journal of Clinical Oncology*.

[51] Chng WJ, Braggio E, Mulligan G (2008). The centrosome index is a powerful prognostic marker in myeloma and identifies a cohort of patients that might benefit from aurora kinase inhibition. *Blood*.

[52] Dickens NJ, Walker BA, Leone PE (2010). Homozygous deletion mapping in myeloma samples identifies genes and an expression signature relevant to pathogenesis and outcome. *Clinical Cancer Research*.

[53] Moreaux J, Klein B, Bataille R (2011). A high-risk signature for patients with multiple myeloma established from the molecular classification of human myeloma cell lines. *Haematologica*.

[54] Hose D, Rème T, Hielscher T (2011). Proliferation is a central independent prognostic factor and target for personalized and risk-adapted treatment in multiple myeloma. *Haematologica*.

[55] Kuiper R, Broyl A, De Knegt Y (2012). A gene expression signature for high-risk multiple myeloma. *Leukemia*.

[56] Chung T-H, Mulligan G, Fonseca R, Chng WJ (2013). A novel measure of chromosome instability can account for prognostic difference in multiple myeloma. *PLoS ONE*.

[57] Chng WJ, Dispenzieri A, Chim CS (2014). IMWG consensus on risk stratification in multiple myeloma. *Leukemia*.

[58] Yates LR, Campbell PJ (2012). Evolution of the cancer genome. *Nature Reviews Genetics*.

[59] Bolli N, Avet-Loiseau H, Wedge DC (2014). Heterogeneity of genomic evolution and mutational profiles in multiple myeloma. *Nature Communications*.

[60] Keats JJ, Chesi M, Egan JB (2012). Clonal competition with alternating dominance in multiple myeloma. *Blood*.

[61] Egan JB, Shi C-X, Tembe W (2012). Whole-genome sequencing of multiple myeloma from diagnosis to plasma cell leukemia reveals genomic initiating events, evolution, and clonal tides. *Blood*.

[62] Tubio JMC, Estivill X (2011). Cancer: when catastrophe strikes a cell. *Nature*.

[63] Magrangeas F, Avet-Loiseau H, Munshi NC, Minvielle S (2011). Chromothripsis identifies a rare and aggressive entity among newly diagnosed multiple myeloma patients. *Blood*.

[64] Walker BA, Wardell CP, Melchor L (2014). Intraclonal heterogeneity is a critical early event in the development of myeloma and precedes the development of clinical symptoms. *Leukemia*.

[65] Chesi M, Robbiani DF, Sebag M (2008). AID-dependent activation of a MYC transgene induces multiple myeloma in a conditional mouse model of post-germinal center malignancies. *Cancer Cell*.

[66] Drewinko B, Alexanian R, Boyer H, Barlogie B, Rubinow SI (1981). The growth fraction of human myeloma cells. *Blood*.

[67] Hamburger A, Salmon SS (1977). Primary bioassay of human myeloma stem cells. *Journal of Clinical Investigation*.

[68] Bakkus MHC, van Riet I, van Camp B, Thielemans K (1994). Evidence that the clonogenic cell in multiple myeloma originates from a pre-switched but somatically mutated B cell. *British Journal of Haematology*.

[69] Pilarski LM, Belch AR (2002). Clonotypic myeloma cells able to xenograft myeloma to nonobese diabetic severe combined immunodeficient mice copurify with CD34+ hematopoietic progenitors. *Clinical Cancer Research*.

[70] Matsui W, Huff CA, Wang Q (2004). Characterization of clonogenic multiple myeloma cells. *Blood*.

[71] Epstein J (1997). Myeloma stem cell phenotype: implications for treatment. *Hematology/Oncology Clinics of North America*.

[72] Gottesman MM, Fojo T, Bates SE (2002). Multidrug resistance in cancer: role of ATP-dependent transporters. *Nature Reviews Cancer*.

[73] Reghunathan R, Bi C, Liu SC (2013). Clonogenic multiple myeloma cells have shared stemness signature assocuated with patient survival. *Oncotarget*.

[74] Rasmussen T, Jensen L, Johnsen HE (2000). The clonal hierachy in multiple myeloma. *Acta Oncologica*.

[75] Boucher K, Parquet N, Widen R (2012). Stemness of B-cell progenitors in multiple myeloma bone marrow. *Clinical Cancer Research*.

[76] Chaidos A, Barnes CP, Cowan G (2013). Clinical drug resistance linked to interconvertible phenotypic and functional states of tumor-propagating cells in multiple myeloma. *Blood*.

[77] Leung-Hagesteijn C, Erdmann N, Cheung G (2013). Xbp1s-negative tumor B cells and pre-plasmablasts mediate therapeutic proteasome inhibitor resistance in multiple myeloma. *Cancer Cell*.

[78] Yang Y, Shi J, Tolomelli G (2013). RARalpha2 expression confers myeloma stem cell features. *Blood*.

[79] Kassambara A, Hose D, Moreaux J (2012). Identification of pluripotent and adult stem cell genes unrelated to cell cycle and associated with poor prognosis in multiple myeloma. *PLoS ONE*.

[80] Paiva B, Vidriales M-B, Cerveró J (2008). Multiparameter flow cytometric remission is the most relevant prognostic factor for multiple myeloma patients who undergo autologous stem cell transplantation. *Blood*.

[81] Rawstron AC, Child JA, de Tute RM (2013). Minimal residual disease assessed by multiparameter flow cytometry in multiple myeloma: impact on outcome in the Medical Research Council Myeloma IX Study. *Journal of Clinical Oncology*.

[82] Sarasquete ME, García-Sanz R, González D (2005). Minimal residual disease monitoring in multiple myeloma: a comparison between allelic-specific oligonucleotide real-time quantitative polymerase chain reaction and flow cytometry. *Haematologica*.

[83] Zamagni E, Patriarca F, Nanni C (2011). Prognostic relevance of 18-F FDG PET/CT in newly diagnosed multiple myeloma patients treated with up-front autologous transplantation. *Blood*.

[84] Matsui W, Wang Q, Barber JP (2008). Clonogenic multiple myeloma progenitors, stem cell properties, and drug resistance. *Cancer Research*.

[85] Peacock CD, Wang Q, Gesell GS (2007). Hedgehog signaling maintains a tumor stem cell compartment in multiple myeloma. *Proceedings of the National Academy of Sciences of the United States of America*.

[86] Chiron D, Maïga S, Descamps G (2012). Critical role of the NOTCH ligand JAG2 in self-renewal of myeloma cells. *Blood Cells, Molecules, and Diseases*.

[87] Brennan SK, Wang Q, Tressler R (2010). Telomerase inhibition targets clonogenic multiple myeloma cells through telomere length-dependent and independent Mechanisms. *PLoS ONE*.

[88] Björkstrand B, Ljungman P, Svensson H (1996). Allogeneic bone marrow transplantation versus autologous stem cell transplantation in multiple myeloma: a retrospective case-matched study from the European Group for Blood and Marrow Transplantation. *Blood*.

[89] Swift BE, Williams BA, Kosaka Y (2012). Natural killer cell lines preferentially kill clonogenic multiple myeloma cells and decrease myeloma engraftment in a bioluminescent xenograft mouse model. *Haematologica*.

